# TiO_2_ nanostructured implant surface-mediated M2c polarization of inflammatory monocyte requiring intact cytoskeleton rearrangement

**DOI:** 10.1186/s12951-022-01751-9

**Published:** 2023-01-02

**Authors:** Zhaoyue Fu, Yongli Hou, Håvard Jostein Haugen, Xutao Chen, Kang Tang, Liang Fang, Yong Liu, Shu Zhang, Qianli Ma, Lihua Chen

**Affiliations:** 1 Department of Immunology, School of Basic Medicine, Fourth Military Medical University, 169 West Changle Road, Xi’an, 710032 People’s Republic of China; 2 Department of Biomaterials, Institute of Clinical Dentistry, University of Oslo, 0317 Oslo, Norway; 3 The Key Laboratory of Aerospace Medicine, Ministry of Education, Air Force Medical University, Xi’an, 710032 Shaanxi China

**Keywords:** TiO_2_, Cytoskeleton, Inflammatory monocyte (iMos), Simulated microgravity (SMG), Macrophage polarization

## Abstract

**Background:**

Microgravity directly disturbs the reorganization of the cytoskeleton, exerting profound effects on the physiological process of macrophages. Although it has been established that macrophage M1/M2 polarization could be manipulated by the surface nanostructure of biomaterial in our previous study under normal gravity, how will inflammatory monocytes (iMos)-derived macrophages respond to diverse nanostructured Ti surfaces under normal gravity or microgravity remains unrevealed.

**Results:**

In this study, Cytochalasin D, a cytoskeleton relaxant, was employed to establish the simulated microgravity (SMG) environment. Our results showed that human iMos polarized into M2c macrophages on NT5 surface but M1 type on NT20 surface with divergent inflammatory phenotypes according to the profile of macrophage polarization featured molecules under normal gravity. However, such manipulative effects of NTs surfaces on iMos-derived macrophages were strikingly weakened by SMG, characterized by the altered macrophage morphology, changed cytokine secretion profile, and decreased cell polarization capacity.

**Conclusions:**

To our knowledge, this is the first metallic implantable material study focusing on the functions of specific monocyte subsets and its crucial role of the cytoskeleton in materials-mediated host immune response, which enriches our mechanism knowledge about the crosstalk between immunocytes and biomaterials. The results obtained in the present study may also provide potential targets and strategies for biomaterial development and clinical treatment via precise immune-regulation under normal gravity and microgravity.

**Graphic Abstract:**

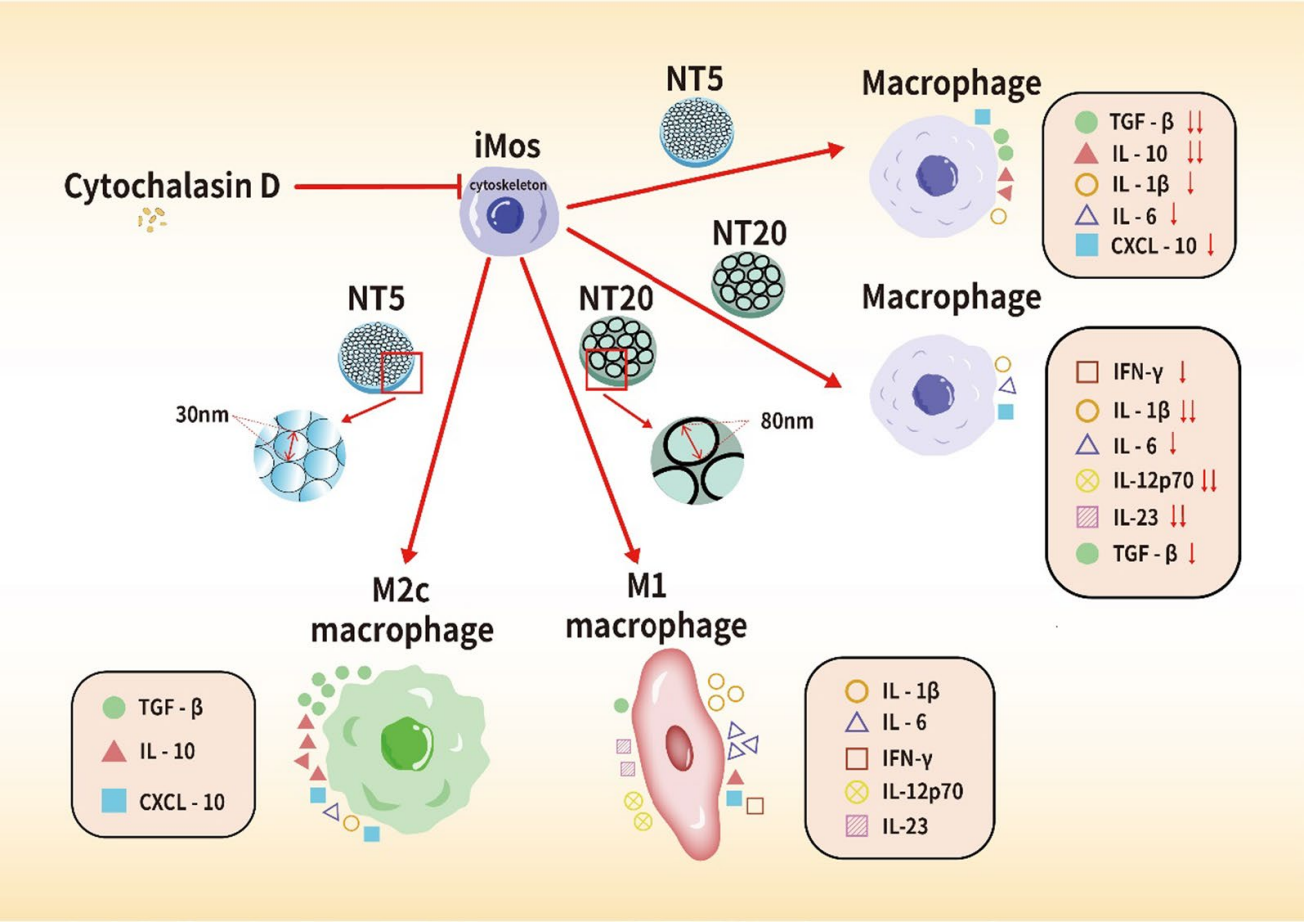

**Supplementary Information:**

The online version contains supplementary material available at 10.1186/s12951-022-01751-9.

## Introduction

Endosseous implantable materials (EIM) have been used extensively in orthopedics and dentistry. The interactions between EIM and cells, such as bone marrow mesenchymal stem cells (bMSCs) and monocytes/macrophages, are responsible for the subsequent progression of bone regeneration. As a crucial determinant of cellular plasticity, phenotypic alteration and survival, the cytoskeleton can be reorganized in response to the stress–strain signals mediated by the chemical-physical characteristics of biomaterial surfaces and thus plays an essential role in the osseointegration of EIM. Moreover, as a sensor of the external environment, the cytoskeletal functions can be strongly affected by microgravity exposure, leading to a decreased expression of actin protein and cell adhesion molecule ICAM-1 accompanied by F-actin depolymerization [1, 2]. Such severely perturbed F-actin reorganization is believed to indicate a phenotypical dysfunction of osteoblastic lineage cells and monocytes/macrophages, and directly interfere with host osteogenesis and immune responses [3–5]. However, most of the current research on implantable materials is conducted under normal gravity or focuses on osteoblastic lineage cells [6, 7]. How monocytes/macrophages respond to nanostructured implantable materials under microgravity is little known.

As the core member of the local innate immune system, macrophages play a key role in the onset, development, and outcome of inflammation, which has been proven to be associated with macrophage M1 and M2 polarization [8]. Our previous work focused on surface nano-topography’s manipulative effects on macrophage's inflammatory responses and found that NT5 induced macrophage M2 polarization whereas NT20 mediated M1 polarization without any exogenous inducers [9, 10]. Such phenotypic alteration of macrophage not only changed the cytokines secretion profile in the microenvironment but also altered the pattern of sRANKL/OPG/M-CSF secretion from bMSCs which directly controlled the osteoclastogenesis and further influenced the balance of bone formation/resorption [11]. Although such findings preliminarily explained the inconsistent osteogenic performances of NT5 and NT20 implants in vitro versus in vivo, the M1/M2 classification of macrophages is too rough and still leads to confusion.

Peripheral circulating monocytes, as the precursors of macrophages, can be divided into subpopulations of classical CD14^+^CD16^-^ monocytes (iMos) and patrolling CD14^lo^CD16^+^ monocytes (pMos) in humans [26], while mouse iMos and pMos are distinguished with CX_3_CR1^int^CCR2^hi^Ly6C^hi^ and CX_3_CR1^hi^CCR2^-^Ly6C^lo^ [12, 13]. iMos account for 85–90% and pMos account for 10–15% of the total number of peripheral monocytes. Heterogeneity also lies in that iMos are recruited to inflammatory sites through the CCR2-CCL2 axis and then further extravasate and differentiate into tissue macrophages and dendritic cells, responding to bacterial and parasitic infections [14], while pMos are localized in the microvasculature of different organs via CX_3_CR1-CX_3_CL1 axis, where they patrol the capillaries, scavenge tissue and cell debris [15]. Unlike iMos, pMos rarely extravasate into the tissue and differentiate into macrophages but can respond to pathological stimuli and contribute to the resolution of inflammation [16–20]. Although the importance of iMos and pMos has received extensive attention in disease research [21], it has not been fully illuminated in implantable materials research. Meanwhile, with significant progress in studying the change of macrophages under microgravity, how will EIM affect iMos-derived macrophages under microgravity is yet to be elucidated. Regarding the similar capacity of inhibiting cytoskeleton reorganization as microgravity, cytochalasin D can be utilized to simulate microgravity-induced cytoskeleton dysfunction. Like microgravity, cytochalasin D was reported to suppress osteogenic differentiation but promote adipogenic differentiation of bMSCs significantly through depolymerization of F-actin [9, 22, 23]. Therefore, it is reasonable to hypothesize that the simulated microgravity (SMG) established by cytochalasin D can similarly manipulate iMos.

In the present study, we purified human peripheral iMos and investigated the inflammatory differentiation of iMos on nanostructured Ti implant surface using analytical methods. According to our hypothesis that SMG will exert the effects of phenotypic and functional remodeling on iMos on NTs surfaces, cytochalasin D treatment was performed in this study. In addition, we reported that iMos were directly induced to differentiate into M2c phenotype on NTs with specific nanotube size (~ 30nm) without exogenous inducers under normal gravity. SMG abrogated such phenotypical alternation, most likely due to the crucial role of cytoskeleton rearrangement in macrophage polarization on sensing the topographical information of biomaterials and the external environment. The results obtained in this study confirm that cytoskeleton plays a crucial role in reprogramming the phenotype of monocyte/macrophage in response to surface nano-topography on biomaterials. As the initial gravity sensor, cytoskeleton can be significantly rearranged under microgravity, which suggests a precise and robust regulative target for immune responses on biomaterials surface under microgravity.

It is well known that although TiO_2_ implants are widely applied in dental implantation and orthopedic surgery [24–26], the innate immune response elicited by the TiO_2_ implant will result in improper osteogenesis, which eventually causes implantation failure [27, 28]. Therefore, to clarify the mechanism of how nanostructured TiO_2_ regulates macrophage polarization is of great importance. Our study preliminarily revealed that the inducing effect of nanostructured TiO_2_ on macrophage polarization was cytoskeleton-dependent, which enlightened us that in the future, we could change the polarization and features of macrophage through modulating cytoskeleton in order to meet the clinical needs. In addition, cytochalasin D was applied in our study to establish simulated microgravity (SMG) and investigate how iMos react to the nanostructure of TiO_2_ under SMG, last but not least which might provide us with experimental evidence for understanding the alterations of iMos on TiO_2_ surface in space. Taken together, our results may provide an intellectual foundation, potential targets of biomaterials development/modification, and clinical treatment strategy via precise immuno-regulation under normal gravity and microgravity.

## Results

### Characterization of nanostructured TiO_2 _surface on Ti implant

**Fig. 1:**
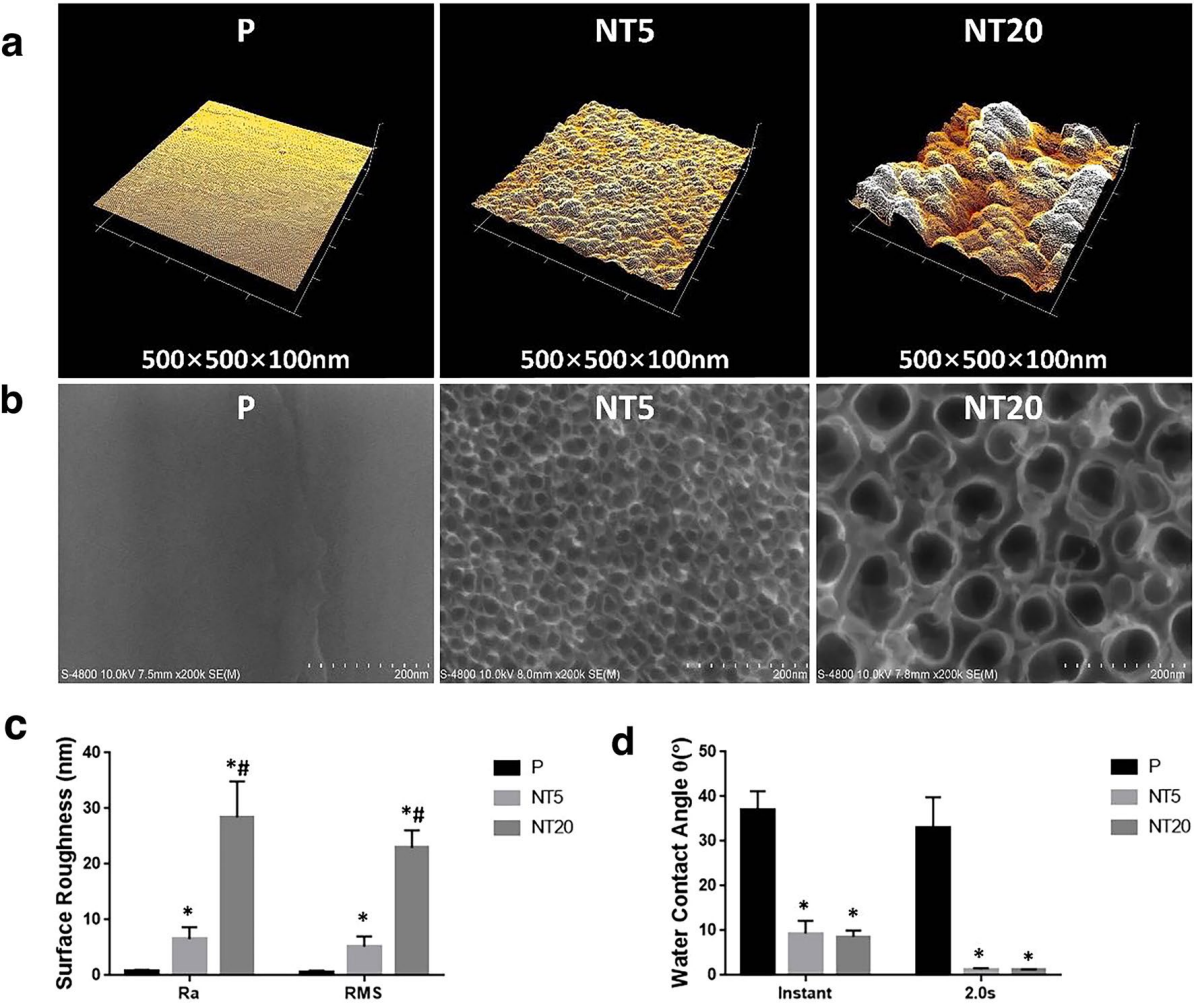
Surface characterization of nanostructured Ti surfaces. **a**, **b** Representative AFM(scan size = 500 μm × 500 μm × 100 μm) and FE-SEM(magnification = 100,000 × ; scale bar = 100 nm) images showing the morphology and topography of nanostructured surfaces(NT5&NT20) and polished Ti controls(P). **c** Quantitive analysis of the roughness of prepared Ti surfaces. **d** Quantitive analysis of hydrophilicity of prepared surfaces (0–2.0 s post contact). *p < 0.01 vs P, #*p* < 0.01 vs NT5. (n = 4, repeated thrice, analyzed using ANOVA)

FE-SEM and AFM conducted the morphological observation and measurement of nanostructured TiO_2_ surface. As shown in Fig. 1b, smooth topography could be observed on P surface while TiO_2_ nanotubes with different tube sizes were distributed uniformly on NTs surface. The tube size was ~ 30 nm on NT5 surface and ~ 80 nm on NT20 surface. The AFM analysis showed that the surface roughness increased with the elevation of anodization voltage (Fig. 1a, c). The hydrophilicity of NTs samples increased significantly compared with P After anodization and UV irradiation. No difference could be seen between NT5 and NT20 (Fig. 1d).

### Behavior of iMos-derived macrophages in the absence of cytochalasin D

#### Cytokine secretions of monocyte/macrophage on nanostructured TiO_2_surfaces

**Fig. 2:**
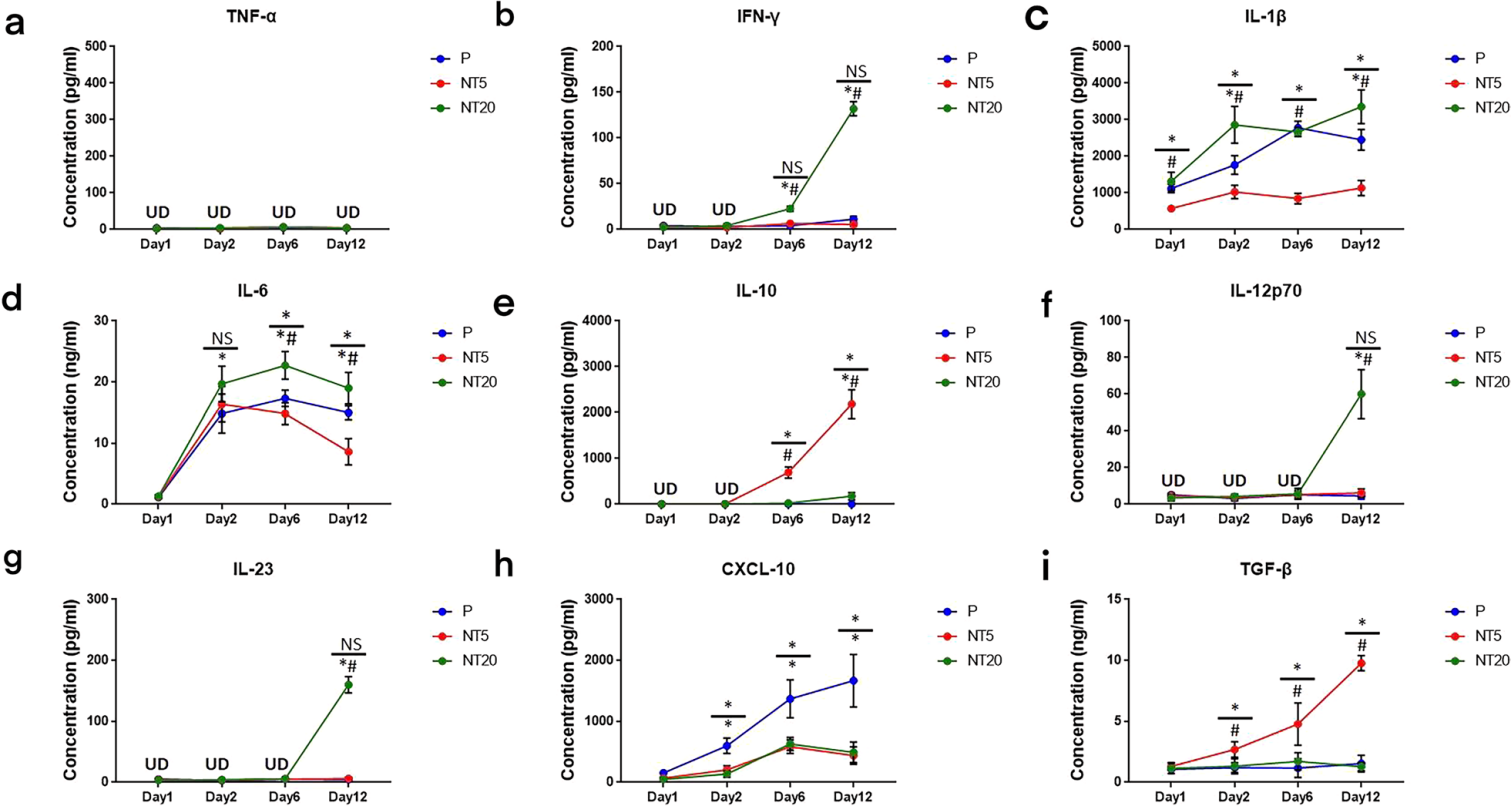
Cytokines secretion profile of human monocytes/macrophages cultured on polished Ti surface (P) and TiO_2_ nanotubular surfaces (NT5 and NT20) for up to 12 days: **a** TNF-α, **b** IFN-γ, **c** IL-1β, **d** IL-6, **e** IL-10, **f** IL-12p70, **g** IL-23, **h** CXCL-10, **i** TGF-β. $\frac{*}{*\#}$ = $\frac{\mathrm{NT}5\mathrm{ vs P}}{\mathrm{NT}20\mathrm{ vs P}\&\mathrm{NT}5}$, *p* < 0.01. (n = 4, repeated thrice, analyzed using ANOVA)

The level of cytokines in supernatants were detected by ELISA assay as shown in Fig. 2. The comparison between different groups can be found in Table. 1. In summary, NT20 surface was prone to inducing secretion of pro-inflammatory cytokines such as IFN-γ, IL-1β, IL-6, IL-12p70 and IL-23 (Fig. 2b–d, f, g) whereas the secretion of anti-inflammatory and regenerative cytokines such as IL-10 and TGF-β on NT5 surface was significantly promoted (Fig. 2e, i). Chemokine CXCL-10 secretion was obviously suppressed on both NTs surfaces and no TNF-α could be detected in all groups (Fig. 2a, h). Intriguingly, such divergence gradually increased with the extension of culture time (Day 12). Flow cytometry and immunofluorescent staining were conducted to specify the polarization of iMos-derived macrophage on nanostructured TiO_2_ surfaces.

**Table 1:** Summary of macrophage cytokine secretions on different surfaces

Phenotype	P	NT5	NT20
TNF-α	−	−	−
IFN-γ	−	−	+
IL-1β	+ +	+	+ + +
IL-6	+ +	+	+ + +
IL-10	−	+ + +	+
IL-12p70	−	−	+ +
IL-23	−	−	+ +
CXCL-10	+ + +	+	+
TGF-β	+ −	+ + + +	+ −

− No secretion; + −, extremely low secretion; + , low secretion; +  + , middle secretion; +  +  + / +  +  +  + , high secretion

#### Characterization of macrophage polarization on nanostructured TiO_2_ surfaces

**Fig. 3:**
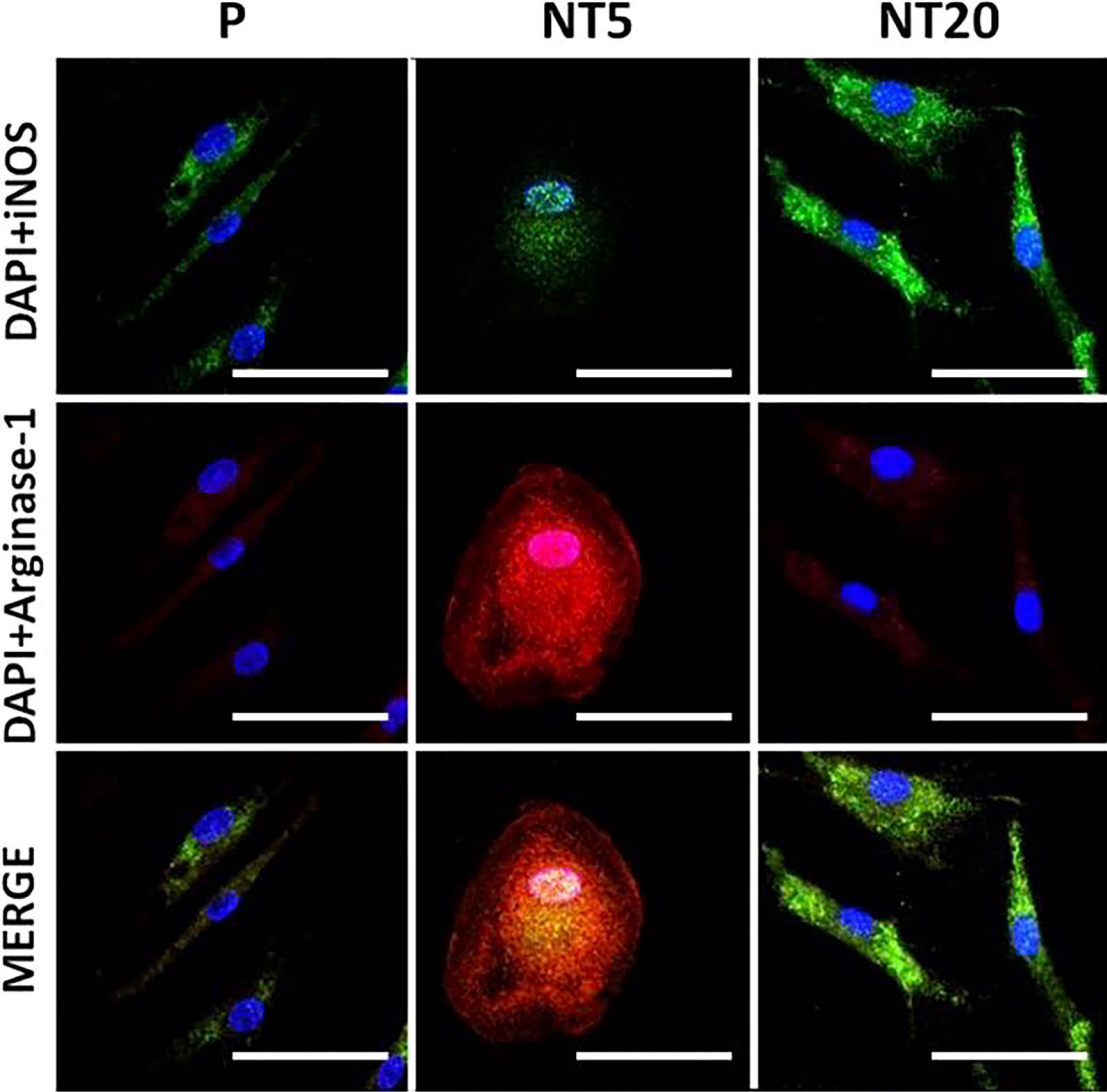
Analysis of macrophage polarization on nanostructured surfaces after 12 days according to immunofluorescent staining assay for iNOS and Arginase-1. Magnification = 600 × ; scale bar = 100 μm. (n = 3, repeated thrice, analyzed using ANOVA)

The different expression of macrophage polarization markers was also verified by immunofluorescent staining, which indicated the elevated iNos signal on NT20 and enhanced Arginase-1 on NT5 surface (Fig. 3). The morphology of macrophages differed a lot between different groups. Macrophages on P and NT20 surfaces obviously stretched while keeping a round shape on NT5.

**Fig. 4:**
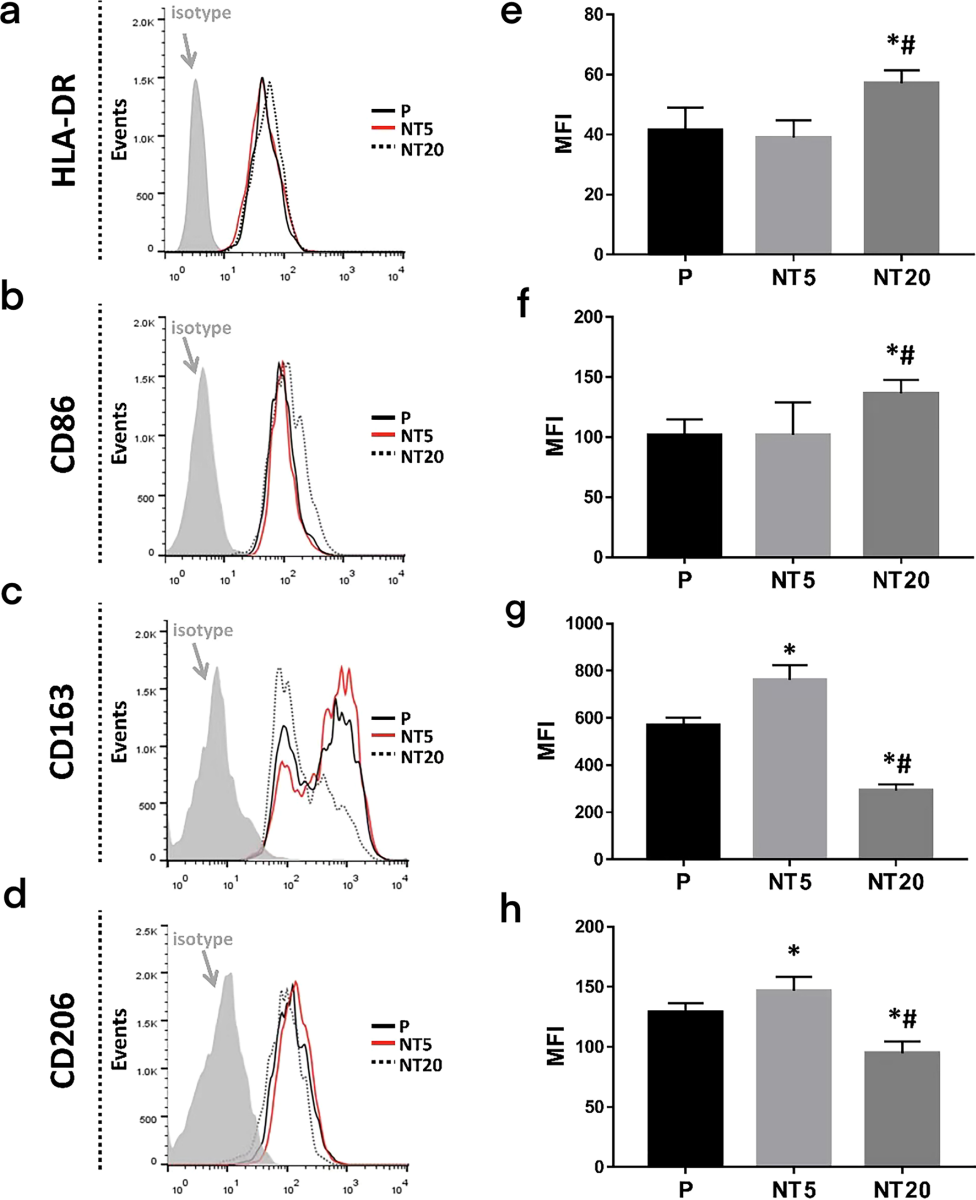
Analysis of macrophage polarization on nanostructured TiO_2_ surfaces after 12 days according to flow cytometry (**a**–**d**) and net MFI measurement (**e**–**h**). *p < 0.01 vs P, #*p* < 0.01 vs NT5. (n = 3, repeated thrice, analyzed using ANOVA)

Besides, to figure out the phenotype of macrophage more specifically, the expression of HLA-DR, CD86, CD163 and CD206 on cell membrane were also inspected. The peak shifting and MFI analysis in Fig. 4 clearly showed that NT20 promoted the expression of HLA-DR and CD86 (M1 marker) while inhibiting CD163 and CD206 (M2 marker) expression. NT5 did not affect HLA-DR and CD86 expression whereas significantly enhanced the expression of CD163 and CD206.

### Behavior of monocytes in the presence of cytochalasin D

#### Cytokine secretions of monocyte/macrophage on nanostructured TiO_2_ surfaces in presence of cytochalasin D

**Fig. 5:**
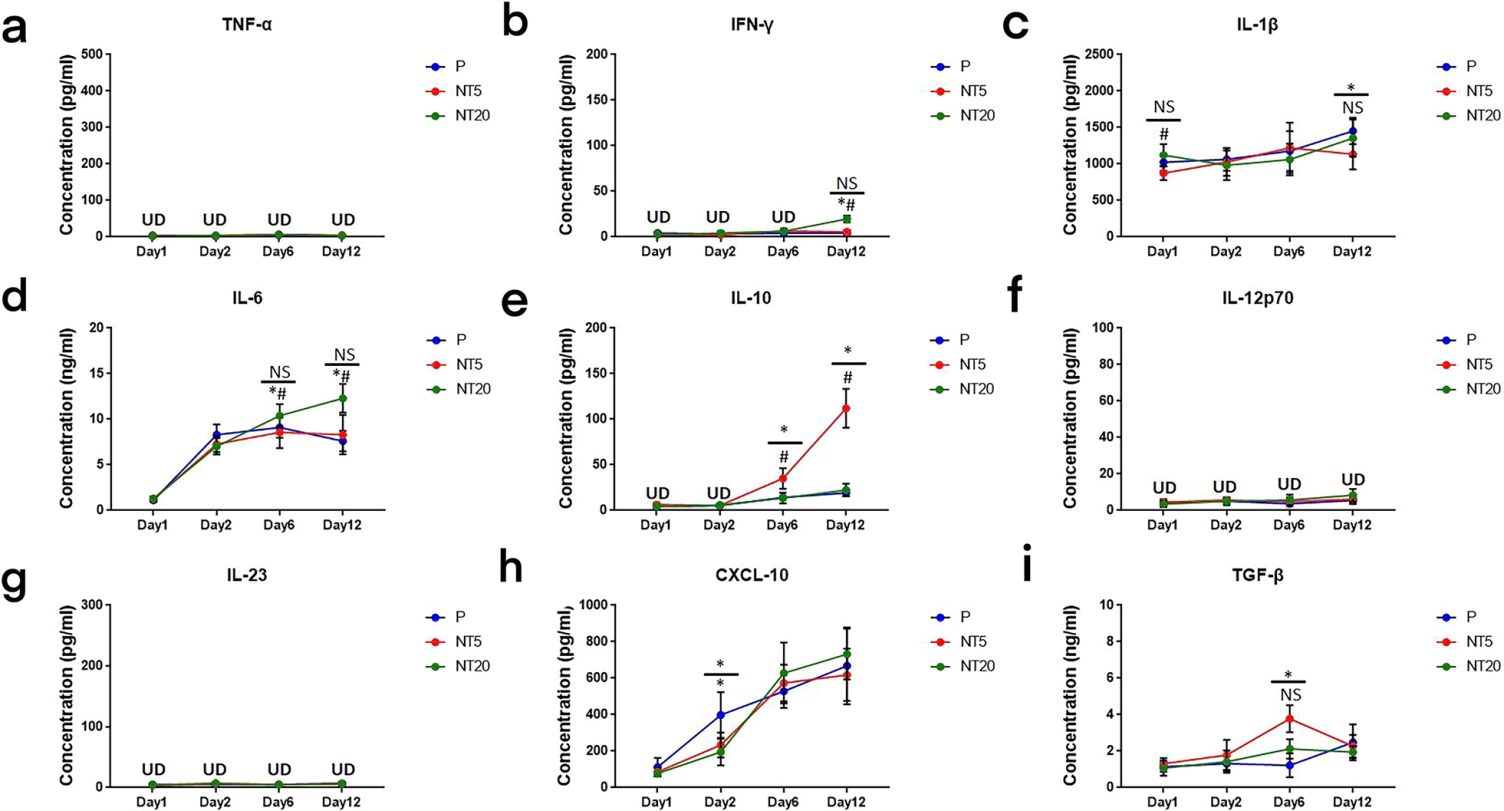
Cytokines secretion profiles of human monocytes/macrophages cultured on polished Ti surface (P) and TiO_2_ nanotubular surfaces (NT5 and NT20) in SMG (Cytochalasin D 200 ng/ml) for up to 12 days: **a** TNF-α, **b** IFN-γ, **c** IL-1β, **d** IL-6, **e** IL-10, **f** IL-12p70, **g** IL-23, **h** CXCL-10, **i** TGF-β. $\frac{*}{*\#}$ = $\frac{\mathrm{NT}5\mathrm{ vs P}}{\mathrm{NT}20\mathrm{ vs P}\&\mathrm{NT}5}$, *p* < 0.01. (n = 4, repeated thrice, analyzed using ANOVA)

**Fig. 6:**
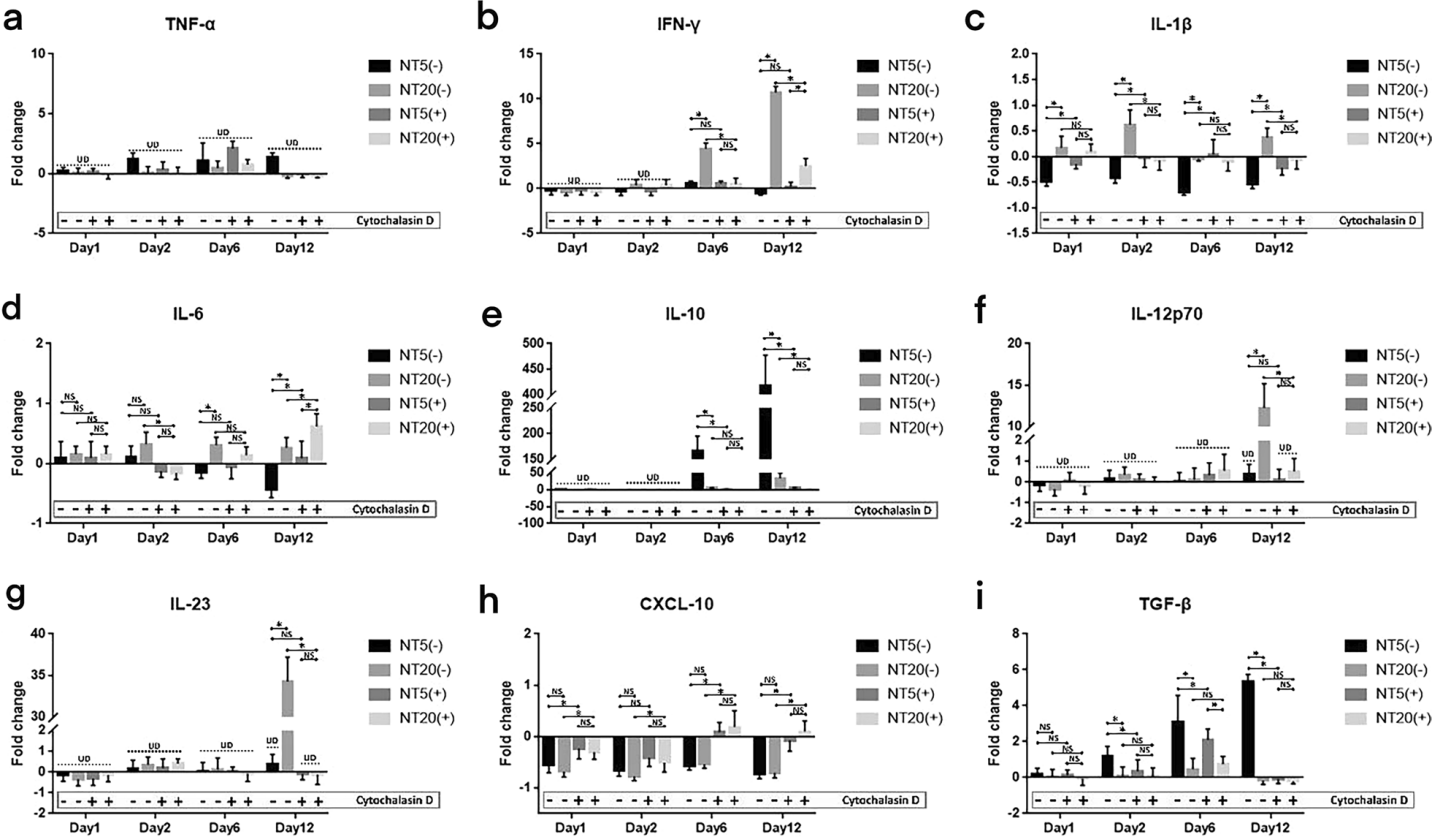
Analysis of cytokines secretion of human monocytes/macrophages cultured on TiO_2_ nanotubular surfaces (NT5 and NT20) compared with their counterpart on polished Ti surface in absence (−)/presence ( +) of Cytochalasin D treatment (Normal gravity/SMG) for up to 12 days: **a** TNF-α, **b** IFN-γ, **c** IL-1β, **d** IL-6, **e** IL-10, **f** IL-12p70, **g** IL-23, **h** CXCL-10, **i** TGF-β. *p < 0.01; *NS* no significance, *UD* under the limit of detection. (Statistical analysis: ANOVA)

In the presence of cytochalasin D (200 ng/ml), the level of abovementioned cytokines was inspected simultaneously. In summary, the secretion of all cytokines was decreased after cytochalasin D treatment (Fig. 5). The secretion of IL-12p70 and IL23 became undetectable and TNF-α kept undetectable as well (Fig. 5a, f, g). The promotive effects of NT20 on IFN-γ, IL-1β, and IL-6 secretion were weakened while the inhibitory effects of NT5 on IL-1β, and IL-6 secretion were also suppressed (Fig. 5b–d). The enhancement of NT5 on IL-10 and TGF-β was largely decreased or even vanished (Fig. 5e, i). Moreover, the cytochalasin D treatment greatly abrogated the suppressive effects of both NTs surfaces on CXCL-10 secretion (Fig. 5h). The fold change analysis of cytokine secretion profile was detailed in Fig. 6.

#### Characterization of macrophage polarization on nanostructured TiO_2_ surfaces in presence of cytochalasin D

**Fig. 7:**
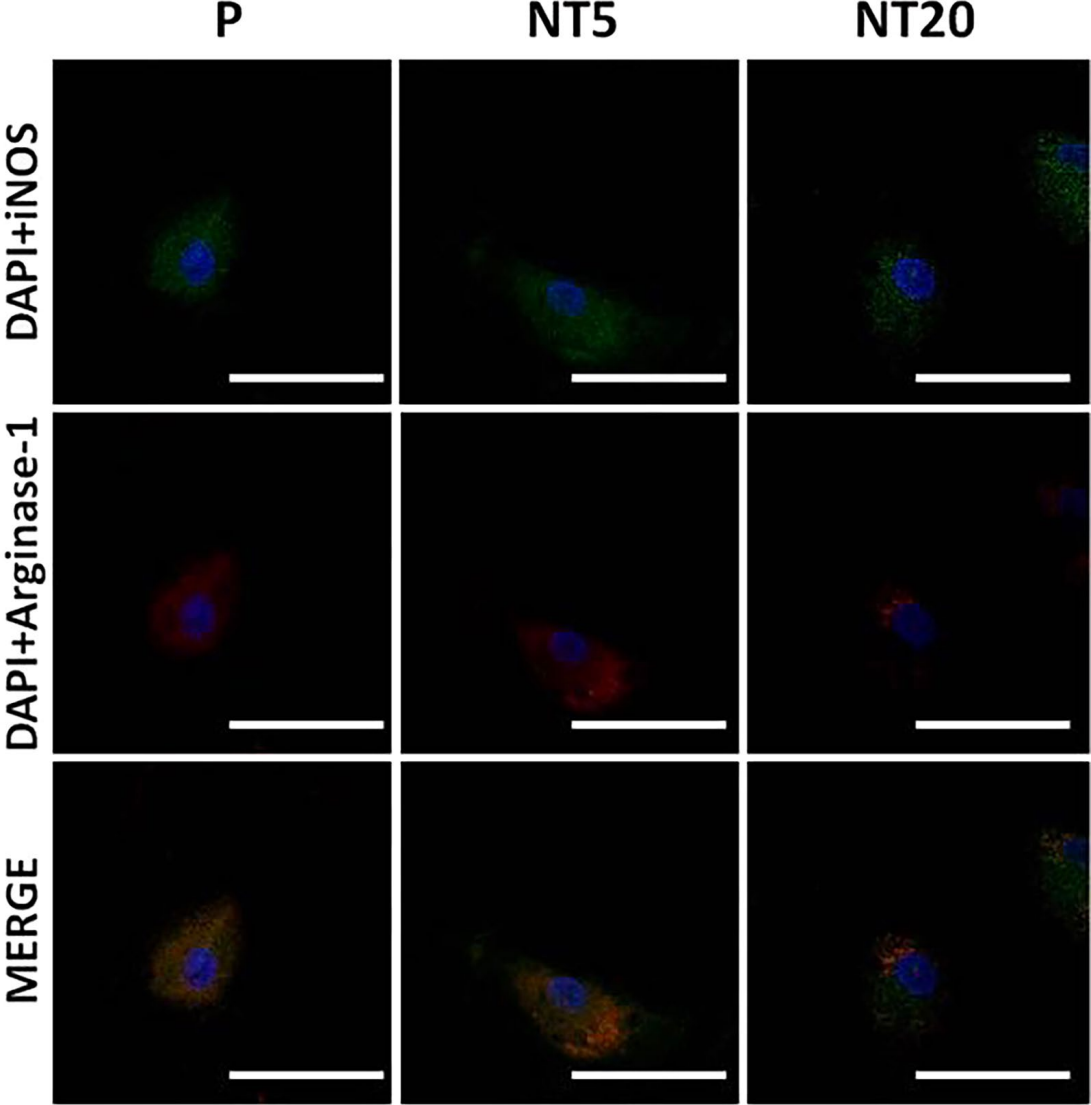
Analysis of macrophage polarization on nanostructured surfaces after 12 days in SMG (Cytochalasin D 200 ng/ml) according to immunofluorescent staining assay for iNOS and Arginase-1. Magnification = 600 × ; scale bar = 100 μm. (n = 3, repeated thrice, analyzed using ANOVA)

As is shown in Fig. 7, the regulatory effects of both NTs groups were invalidated by cytochalasin D treatment. It is worth noting that the morphology of macrophages changed a lot on all three groups. Both polarized stretching and round spreading of macrophage vanished. In situ, the immunofluorescent staining of iNos and Arginiase-1 supported the hampered regulatory effects of TiO_2_ nanostructured surfaces, and no clear polarization of Macrophages could be identified on NTs surfaces.

**Fig. 8:**
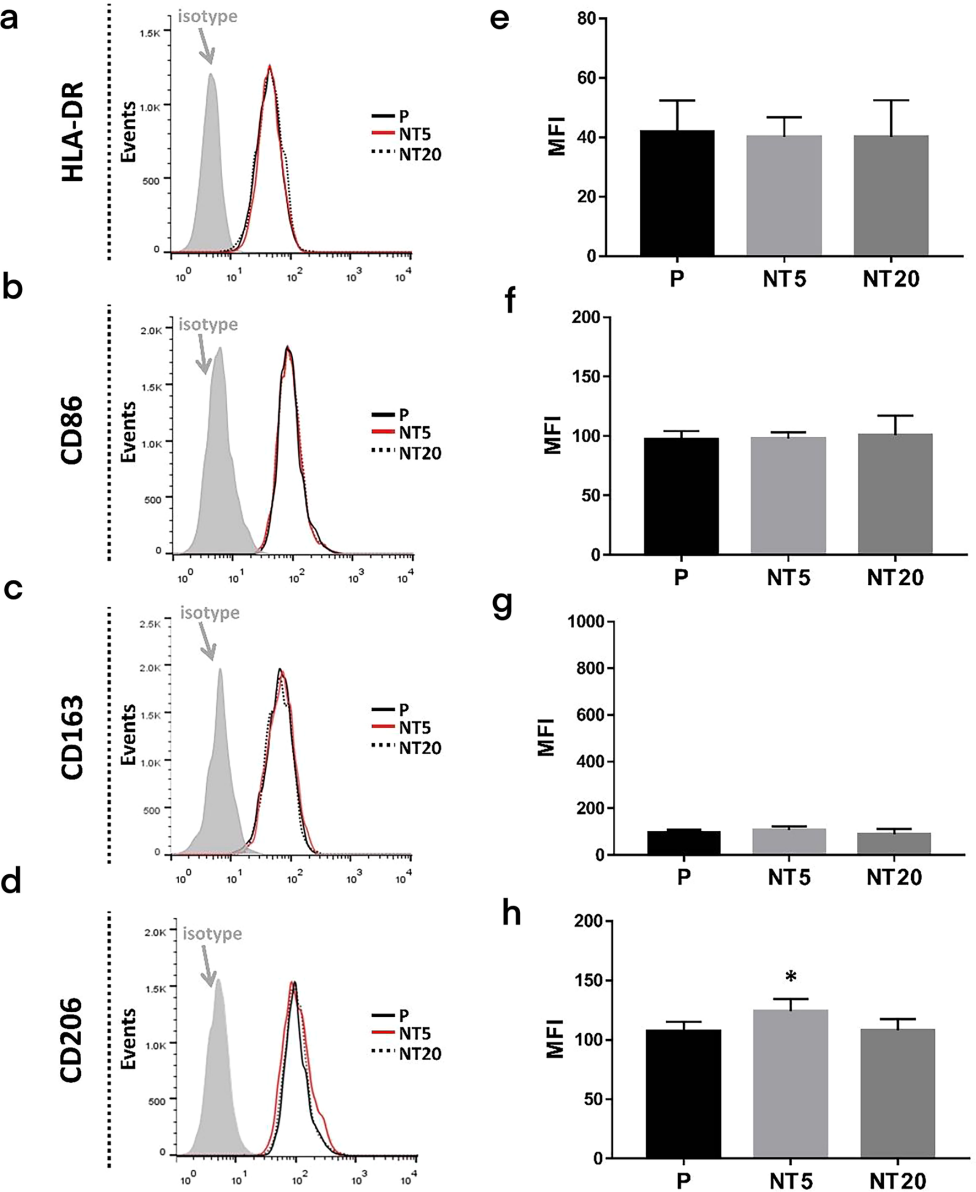
Analysis of macrophage polarization on nanostructured surfaces after 12 days in SMG (Cytochalasin D 200 ng/ml) according to flow cytometry (**a**–**d**) and MFI measurement (**e**–**h**). *p < 0.01 vs P, #*p* < 0.01 vs NT5. (n = 3, repeated thrice, analyzed using ANOVA)

**Fig. 9:**
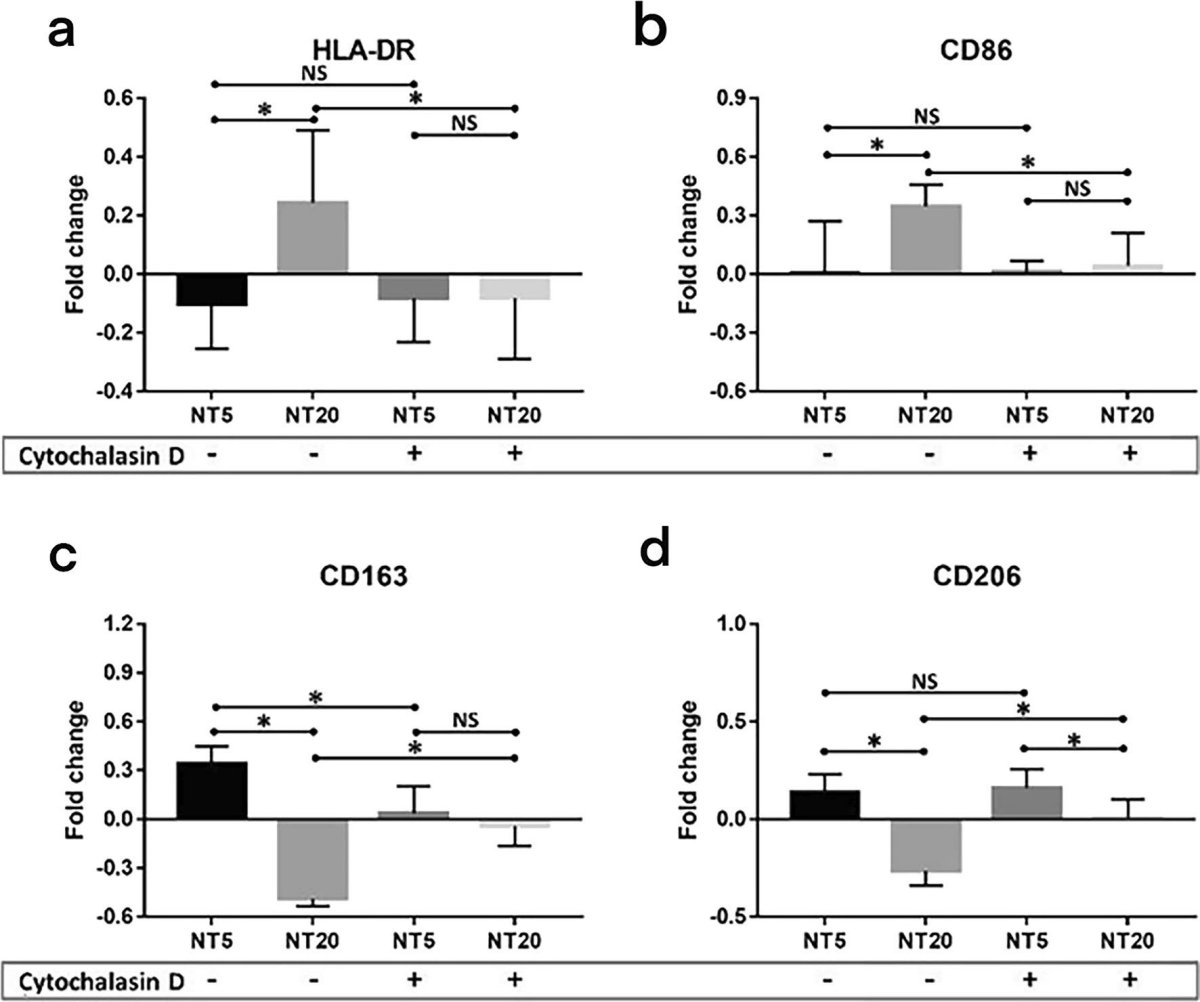
Analysis of macrophage polarization cultured on TiO_2_ nanotubular surfaces (NT5 and NT20) compared with their counterpart on polished Ti surface after 12 days in absence (−)/presence ( +) of Cytochalasin D treatment (Normal gravity/SMG) according to flow cytometry and MFI measurement: **a** HLA-DR, **b** CD86, **c** CD163, **d** CD206. *p < 0.01; NS, no significance. (Statistical analysis: ANOVA)

Similarly, no noticeable difference of HLA-DR, CD86, CD163 expression (Fig. 8a–c and e–g) on different nanostructured TiO_2_ surfaces could be observed whereas NT5 still facilitated CD206 expression of macrophages (Fig. 8d, h). The fold change analysis of the expression of abovementioned molecules was plotted in Fig. 9.

## Discussion

It’s well acknowledged that cells will interact with the nanostructured Ti surfaces selectively depending on their lineages [29]. Besides the regulatory functions on host osteogenic activities, the nano-modified Ti implant surfaces manipulated the M1/M2 polarization of macrophages and related host innate inflammatory responses as well [9, 30]. The polarized macrophage could further interact with bMSCs and collectively decide the balance of osteogenic/osteolytic microenvironment in a mutual feedback manner [11]. Such cell–cell-biomaterials interactions determine the performance and prognosis of implantable materials. However, most previous research model on macrophage behaviors in the presence of biomaterials was based on pan peripheral monocytes and the polarized macrophages were roughly divided into M1 and M2 phenotype [9, 30]. Regarding the fact that peripheral monocytes consisted of at least two subsets: iMos and pMos [12, 13], it is of most importance to figure out the main contributor to implant-related immune response and detailed subsets of macrophage polarization (Additional file 1:Fig. S1).

Alternative perspectives were provided about the subsets of peripheral monocytes. Besides acknowledged iMos (CD14^hi^CD16^−^_,_ also known as classic monocytes), the pMos could further be divided into intermediate monocytes (CD14^hi^CD16^+^) and non-classic monocytes (CD14^+^CD16^hi^) separately [31, 32]. However, it is worth noting that non-classic monocytes only account for less than 10% of all peripheral monocytes [31], and intermediate monocytes were proved most possible to be the intermediate stage between classic and non-classic monocytes [33]. Therefore, the pMos mentioned here referred to intermediate monocytes and non-classic monocytes because of the linear trajectory from classic monocytes to non-classic monocytes [34]. In this study, regarding to the ratio of purified iMos/pMos (7.55 ± 1.24: 1) and OCs formation experiments (Additional file 2: Fig. S2), iMos (~ 1078 OCs/100,000 panMos/1.0 cm^2^) were supposed to be the main source of OCs formation rather than pMos (~ 27 OCs/100,000 panMos/1.0 cm^2^) according to their original density. A previous study also supported such a conclusion, which proved that only iMos-derived OCs possess bone resorptive ability [35]. Besides, the secretion of IL-1β and IL-6 provided further evidence that the static inflammatory functions of iMos were higher than pMos (Additional file 2: Fig. S2c, d). However, under infectious status, iMos exhibited both pro- and anti-inflammatory phenotypes whereas pMos preferred to act as a stubborn inflammation promoter [36, 37]. In addition, lacking of chemokine receptor CCR2 prevent pMos from chemotactic migrating into tissue traumatic site post-surgery. By contrast, iMos represent the first chemoattracted monocytes instantly after implantation. Their attachment on implant surface and subsequent maturation/differentiation can better simulate materials-mediated innate inflammation naturally without covering the flexible inflammatory potential of iMos and the regulatory effects of biomaterials surface nanostructure unexpectedly [9]. Moreover, regarding to the predominant peripheral occupancy, flexible inflammatory potential and sensitivity to environmental signals, iMos were emphasized and involved in the research model of present study.

Generally, titanium and titanium alloys are considered ideal well-performing metallic materials for implantation because of the highly biocompatible TiO_2_ layer on the implant surface [38]. However, the TiO_2_ surface on Ti implant is not completely exempt from inflammatory responses. The phenotypic alteration of macrophages was also crucial for the prognosis of implantation treatment. Our previous work had already proved that the titania nanotubular surface directly manipulated the M1/M2 polarization of human pan monocytes, and further modulated osteogenic/osteolytic balance around Ti implant [9, 11]. Unfortunately, as a broad spectrum, the rough classification of M1/M2 is not enough to describe the nuances of macrophages on NTs surfaces. According to the detailed information in Tables 1 and 2 [39, 40], iMos on NT20 surface were confirmed to polarize into M1 phenotype and iMos on NT5 surface were observed to differentiate into M2c phenotype regarding to the high level of IL-10 and TGF-β. Intriguingly, although some cytokines (IFN-γ, IL-10, IL-12p70 and IL23) were secreted following the typical M1/M2 (a, b, c) patterns, TNF-α were undetectable in NT20 group while the level of IL-1β as well as IL-6 were still considerable in NT5 group. The expressions of HLA-DR, CD86, CD163 and CD206 of NT5 and NT20 groups were also in higher/lower patterns rather than the typical yes/no patterns. The partially overlapped expression of polarization markers also reflected the remarkable plasticity and mutual transformation of M1/M2 macrophages on nanostructured surface [39, 41]. The inflammatory phenotypic alteration is typically believed to be linked to the morphological change of macrophages. McWhorter et al. previously reported that the LPS/IFN-γ induced M1 mouse macrophage kept a round shape, and IL-4 induced M2 macrophage stretched to a spindle-like shape [42]. On the contrary, Pergola et al. reported the stretched human M1 macrophages and round-shaped M2 macrophages induced by LPS/IFN-γ and IL-4, respectively, which was consistent with our study (Fig. 3) but with some detailed differences [43]. The difference in macrophage species (mouse vs human), inducers (cytokines vs blank media) and substrates (pluronic artificial matrix vs nanostructured TiO_2_) may significantly contribute to such divergence and further demonstrates that surface nanostructure may control the shape and activity of macrophages in a different way [44].

**Table 2:** Properties of different polarized macrophages

Phenotype	M1	M2a	M2b	M2c
Inducers	LPS/IFN-γ	IL-4/13	IC	IL-10
CD86	+ +	+ + +	−	+
HLA-DR	+ +	+ + +	−	+
CD163	−	−	−	+ + +
CD206	+	+ + +	−	+ +
TNF-α	+ +	−	+ +	−
IFN-γ	+ +	−	+ +	−
IL-1β	+ +	−	+ +	−
IL-6	+ +	−	+ +	−
IL-10	+	+ + + +	+ + + +	+ + + +
IL-12	+ + + +	−	+	−
IL-23	+ +	−	−	−
TGF-β	−	−	−	+ +

− No expression; +  low expression; +  +  middle expression; +  +  +  high expression

Sharing similar chemical composition, protein absorption and high hydrophilicity as shown previously (Fig. 1d) [9], the topological difference between NT5 and NT20 surfaces is supposed to be the primary environmental cue leading to the divergent polarization of macrophages, which may attribute to the mechanical stress/tension formulated by nanostructures [45, 46]. We hypothesize that the interaction between monocytes and biomaterials begins with integrins-mediated cell adhesion. Once adherent, the integrins on monocytes recognize the nano-topographic information on the implant surface and additional structural proteins (i.e., Vinculin, Paxillin, Talin, and Src. etc.) will be recruited and assembled to form focal adhesions following the pattern of surface nanotexture, which serve as the mechanosensory to sense and deliver tension signals to the nucleus through the actin filaments network [28]. In addition, Rho-family GTPases, such as RhoA, Rac1, Ras and Cdc42, have been primarily proved associated with F-actin rearrangement and following activation of MAPK pathways [47, 48]. He YD et al. previously reported the elevated p-ERK1/2 and p-JNK expression in M1 polarized mouse macrophages on NT20 surface, which could be suppressed by p-FAK inhibitor [44]. The NT5 was recently reported to inhibit mouse osteoclasts formation via suppressing the expression of integrin β1 and p-FAK [49].

With well-characterized structural features, macrophages change their morphological structures according to the external environment and their functional roles. Under normal gravity, activated M1 macrophages have more lamellipodia and filopodia, whereas M2 macrophages possess a rounded structure with actin located primarily around the nucleus [43, 50]. Notably, the long-term (> 72 h) microgravity leads to a decreased expression of actin protein and cell adhesion molecule ICAM-1 accompanied by cytoskeletal disorganization [1, 2]. Such greatly disturbed actin cytoskeleton reorganization is believed to indicate a phenotypical dysfunction of macrophages and directly modulate inflammation-associated gene expression [3–5]. However, disparate use of numerous cell types (primary vs cell line, mouse vs human), polarization inducers and platforms (real space flight vs simulating model) when assessing microgravity make direct comparisons difficult, and no consensus has been formed on the inflammatory secretion as well as polarization of macrophage experienced microgravity [1, 51–54]. Considering the high cost of real microgravity platforms and the central role of cytoskeleton in macrophage polarization, we simplified the simulated microgravity (SMG) platform with Cytochalasin D treatment. As is shown in Additional file 3: Fig. S3, even short-term (12 h) treatment of Cytochalasin D could lead to F-actin depolymerization, accompanied by irregular, softened cell shape and partially overlapped boundaries of macrophage**.** Such SMG system provided us a low-cost and stable platform to reach the repeatable flaccid state of the cytoskeleton, which is quite similar as “real microgravity” studies reported previously.

As is shown in Figs. 2, 3, 4, the M1 and M2c inductive effects of NT20 and NT5 on iMos were observed, respectively. This is the first time for us to figure out the subtype of polarized M2 macrophages on the nanostructured implant surface. However, as a detailed comparison shown in Figs. 6 and 9**,** both pro-/anti-inflammatory functions and M1/M2c manipulative functions of NTs were strikingly weakened or fully abrogated by SMG (Figs. 7 and 8). Meanwhile, macrophages maintained relatively small and round shape on all tested surfaces (Fig. 7 and Additional file 3: Fig. S3, similar as original iMos), instead of the stretched M1 macrophages on NT20 and enlarged, rounded M2 macrophages on NT5 (Figs. 3 and 7), which indicated that intact cytoskeleton functions are crucial for the nanostructured materials-mediated phenotypic alteration of monocytic lineage cells. Furthermore, although SMG could reduce the overall secretion level of most cytokines, it seems impossible to eliminate all cytokines secretion by SMG due to the high baseline level of specific inflammatory cytokines (e.g. IL-1β and IL-6. Etc.). Hence, it is not advisable to abrogate all the material-mediated inflammatory responses by interfering with cytoskeleton rearrangement. The ultimate aim of immune-regulation of biomaterials was to facilitate tissue regeneration by utilizing host inflammation rather than completely avoiding host inflammation. An ideal strategy for materials-mediated precise immune-regulation should be to actively manipulate the M2c polarization of macrophages by utilizing intact cytoskeleton functions. Such materials-induced M2c macrophages will rapidly lead to cascaded M2c polarization of subsequently immigrated monocytes by IL-10 and TGF-β secretion, thereby skewing the osteogenic/osteolytic balance via an amplified M2c predominant microenvironment. However, microgravity will severely perturb the cytoskeletal functions of cells, not only monocytic lineage cells, but almost all other tissue structural cells (e.g. osteoblasts, osteocytes, periosteal cells, bMSCs, fibroblast-like cells etc.), and thus largely invalidates the bio-regulatory functions of biomaterials and directly impairs the host osteogenesis as well [55–58]. Therefore, facing the challenge of microgravity, the top priority is to restore the integrity of cytoskeletal functions, thereby maintaining cellular responses to the designed regulatory functions of biomaterials.

## Materials and methods

### Fabrication and characterization of nanostructured Ti samples

Ti samples with diverse nanostructures were manufactured via a sophisticated process as manifested in our previous study [9]. In brief, pure Ti circular disk samples (99.9%, Grade 1, 14.5 mm in diameter and 1 mm in thickness, for cell culture) were obtained from Northwest Institute for Nonferrous Metal Research (Xi'an, China) and were hierarchically polished with SiC sandpaper (1,500–8,000 grit; Matador, Germany) followed by ultrasonic cleaning with acetone, ethanol, and deionized water in sequence for 5 min each round. Ti samples were then anodized in an aqueous electrolyte solution containing 0.278% (wt %) hydrofluoric acid and 1 M phosphoric acid at 20 °C for 1 h. A direct current power supply and a platinum cathode set at 5 and 20 V were used to fabricate the nanostructured surfaces denoted NT5 and NT20, respectively. Merely polished Ti samples were labeled as P. The topographic analysis of the prepared Ti samples was performed by field emission scanning electron microscopy (FE-SEM; S-4800, Hitachi, Japan) and atomic force microscopy (AFM; Dimension Icon, Bruker, Germany). The contact angles and hydrophilicity of the samples were assessed by a Contact Angle Meter (DSA1 System, Kruss, Germany). The contact angles were measured instantly and 2.0 s after placing a droplet of water with 10 μl of each click with the Drop Shape Analysis (DSA) software. Ti samples were sterilized by UVA/C irradiation (λ = 365 nm (UVA)/254 nm (UVC); Philips, Poland) at a distance of 50 mm for 1 h and then pre-placed in 24-well plates (Nunc, USA) prior to cell culture.

### Human iMos isolation and cultivation

As detailed previously, human monocytes were separated and refined from 10 healthy blood donors [9]. First, ethylenediamine tetra-acetic acid (EDTA)-pretreated buffy coat was obtained from the Xi’jing Hospital, Air Force Medical University's blood bank and diluted 1:1 with RPMI 1640 medium (Cellgro, Corning, USA). 25 ml diluted blood was layered over 15 ml Lymphocyte Separation Medium (LSM, MP, USA) in a 50-ml conical polystyrene tube (Corning, USA) for centrifugation at 450 ×*g* for 25 min continuously at room temperature without brake. The PBMCs in the interphase band (cloudy layer) were collected, resuspended, and washed three times in cold phosphate-buffered saline (PBS; Cellgro, Corning). In the second step, PBMCs were treated with CD16^+^ Monocyte Isolation Kit (Miltenyi Biotec, Germany) and CD14^+^ microbeads (Miltenyi Biotec, Germany) in sequence to deplete CD16^+^ pMos and obtain purified CD14^+^CD16^−^ iMos. Trypan blue staining was performed to determine the cell vitality. Only with the vitality over 95%, iMos can be used for further research. Fresh iMos were resuspended at 1 × 10^6^ cells/ml in α-Minimal Essential Medium (α-MEM, Gibco, USA) containing 5% fetal calf serum (FCS, Gibco, USA) and 1% penicillin–streptomycin (Cellgro, USA) and then planted on the prepared Ti samples (1 × 10^6^ cells/sample) and cultured routinely (humidified atmosphere of 5% CO_2_ at 37 ℃) in presence/absence of 200 ng/ml Cytochalasin D (Sigma-Aldrich, USA).

### Cytokine secretions of monocyte/macrophage on nanostructured titanium surfaces

After 1, 2,6 and 12 days culture, the media were collected and the concentrations of TNF-α, IFN-γ, IL-1β, IL-6, IL-10, IL-12p70, IL-23, CXCL-10 and TGF-β were detected with enzyme-linked immunosorbent assay (ELISA) kits (R&D Systems and Invitrogen, USA) according to the manufacturers’ instructions.

### Flow cytometry (FCM) to detect the polarization of macrophages

After 12 days of culture, iMos-derived macrophages (IDMs) on prepared Ti samples were treated with Accutase® solution (Sigma-Aldrich, USA) for 10 min. After then, IDMs were fully removed by repeated pipetting and cell scraper and washed with FCM wash (PBS with 0.1% BSA). Protein analysis for macrophage polarization was performed using the FACS Aria II system (BD Biosciences, USA) with antibodies specific for surface markers (CD86, HLA-DR, CD163, CD206), which were detailed in Table. 2. The correspondingly IgG isotypes were also applied, and the net Mean fluorescent intensity (MFI) values were analyzed using FlowJo V10.0 software (Tree Star, USA) with the subtraction of MFI_Isotypes_.

### Immunofluorescent staining assay to observe macrophage polarization

As depicted in “Behavior of iMos-derived macrophages in the absence of cytochalasin D” section, iMos were cultivated on different Ti samples and cultured for 12 days. The samples were fixed in 4% paraformaldehyde aqueous solution for 10 min. Samples were then treated with 0.02% TritonX-100/PBS solution for 20 min and 1% BSA/PBS blocking solution for 1 h in sequence. Immunofluorescence staining was performed with mouse anti-iNos and goat anti-Arginase 1 primary antibodies (NovusBio, USA) as well as corresponding NL493- and NL557-conjugated secondary antibodies (R&D systems, USA). After glycerol mounting, samples were observed by LSCM (FV1000, Olympus, Japan).

### Statistics analysis

Experiments were conducted three times to ensure credibility in each group. All statistical analysis were carried out on SPSS 19.0 (IBM, USA) and plotted with Prism 8.0 (GraphPad Software, USA). All data were expressed as the mean ± standard deviation for continuous variables. Significant differences between groups were confirmed using one-way analysis of variance (ANOVA) followed by a Student–Newman–Keuls post hoc test for parametric data or Kruskal–Wallis test followed by a Dunn’s multiple comparison test for non-parametric data. Differences were considered statistically significant when p ≤ 0.01.

## Conclusions

In summary, NT5 surface (tube size ~ 30 nm) can induce human iMos differentiating into anti-inflammatory M2c macrophages, whereas NT20 surface (tube size ~ 80 nm) mediate M1 polarization. SMG inhibited F-actin polymerization, thereby invalidating the manipulative effects of the nanostructured Ti implant surface. This is the first metallic implantable material study, to our knowledge, that is focusing on the functions of specific monocyte subsets and the key role of the cytoskeleton in materials-mediated host immune response, which enriches our mechanism knowledge of the crosstalk between immunocytes and biomaterials. The simplified SMG platform provides a low-cost solution for studying the performance of implantable materials in space environment. The results obtained in the present study may also provide potential interventional targets and strategies for improving the response of cells to nanomodified biomaterials in microgravity.

## Supplementary Information

**Additional file 1: Fig. S1.** Characteristics of human peripheral blood iMos (top row) and pMos (bottom row): iMos and pMos were stained with antibodies of FITC-CD14, PE-CD16, PerCP/Cy5.5-CX_3_CR1 and APC-CCR2.

**Additional file 2: Fig. S2.** The OCs differentiation and inflammatory potential of iMos and pMos. (A) Representative images of iMos/pMos-derived OCs with TRAP and Hoechst33342 staining; (B) Counts of OCs (TRAP+, >3 nuclei) each imaging field in 96-well plate; (C) IL-1βand (D) IL-6 secretion from iMos and pMos after 24h culture; *p<0.05, **p<0.01, ***p<0.001, ****p<0.0001; Scale bar=200 μm. (Statistical analysis: Mann-whitney U test for B, n=60, repeated thrice; ANOVA for C and D, n=3, repeated thrice).

**Additional file 3: Fig. S3.** The F-actin staining (FITC-Phalloidin) of iMos. Left panel: iMos in absence of Cytochalasin D; Right panel: iMos in presence of Cytochalasin D (200 ng/ml) after 12 h. Scale bar=25 μm.
