# Supplementary figures and images for: TiO2 nanostructured implant surface-mediated M2c polarization of inflammatory monocyte requiring intact cytoskeleton rearrangement
Source: J Nanobiotechnology. 2023 Jan 2;21:1. doi: 10.1186/s12951-022-01751-9 (PMC9809010; doi:10.1186/s12951-022-01751-9)

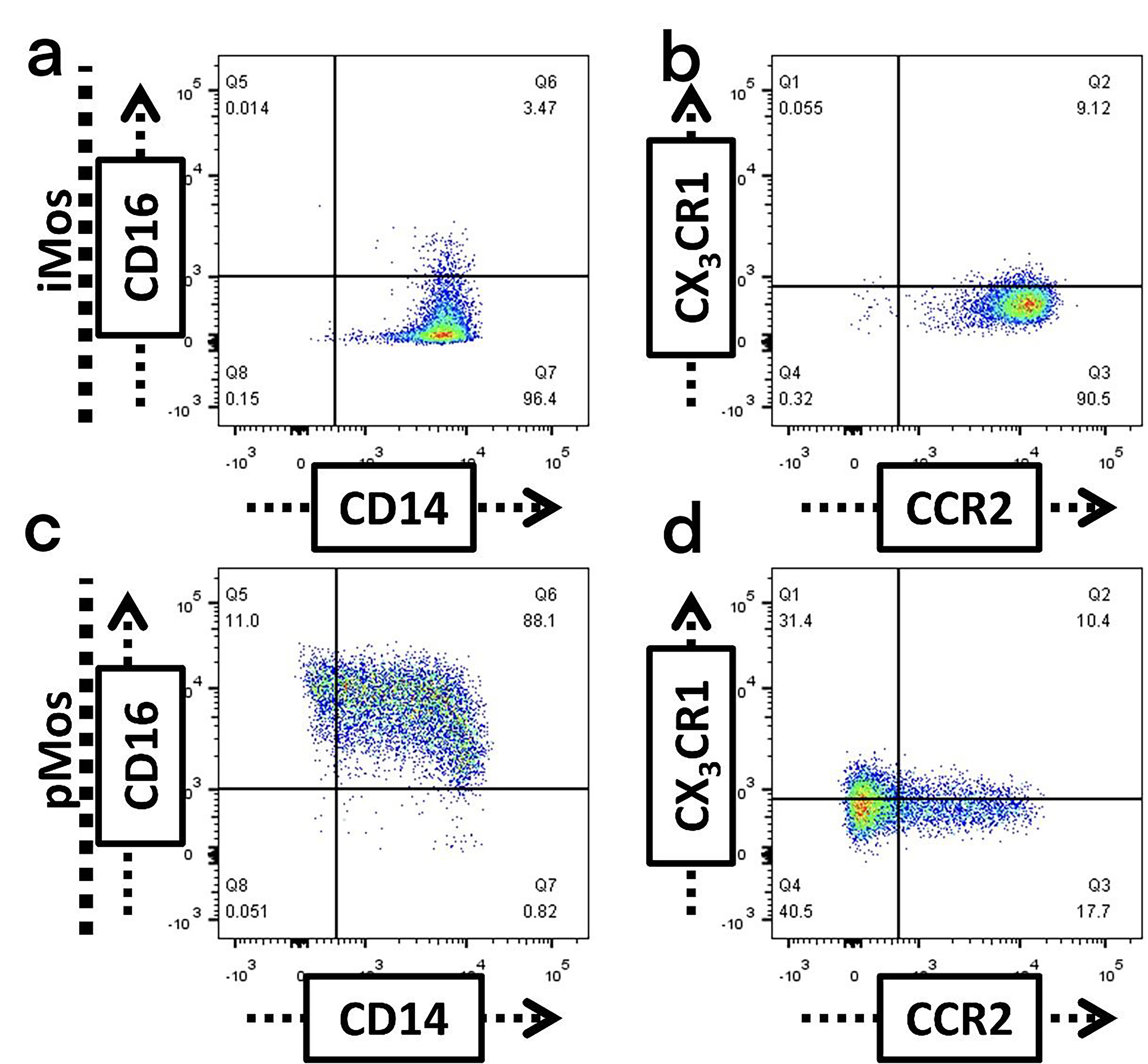

Supplement: Supplementary file 1 — Additional file 1: Fig. S1. Characteristics of human peripheral blood iMos (top row) and pMos (bottom row): iMos and pMos were stained with antibodies of FITC-CD14, PE-CD16, PerCP/Cy5.5-CX3CR1 and APC-CCR2. [file 12951_2022_1751_MOESM1_ESM.jpg]

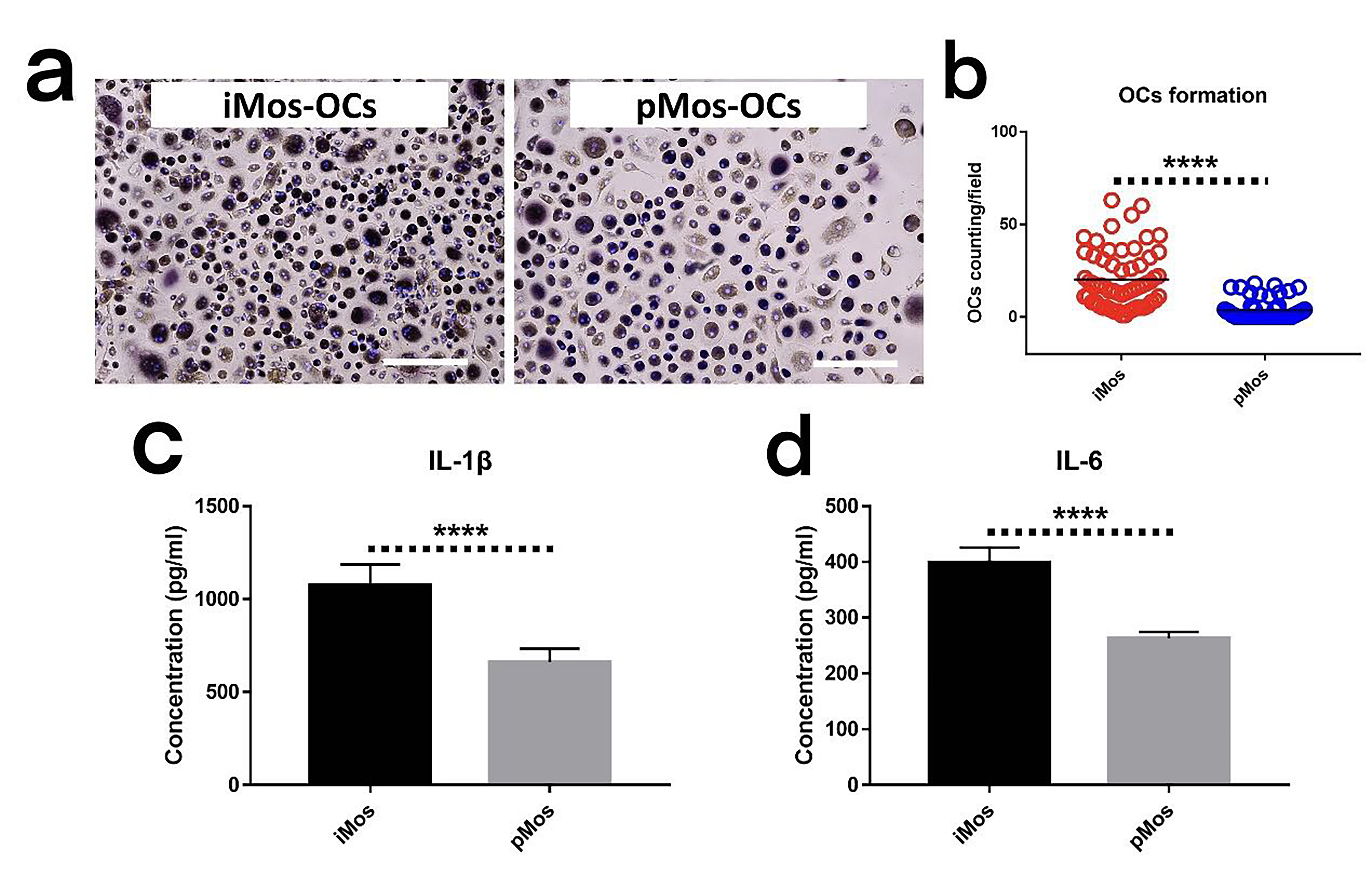

Supplement: Supplementary file 2 — Additional file 2: Fig. S2. The OCs differentiation and inflammatory potential of iMos and pMos. (A) Representative images of iMos/pMos-derived OCs with TRAP and Hoechst33342 staining; (B) Counts of OCs (TRAP+, >3 nuclei) each imaging field in 96-well plate; (C) IL-1βand (D) IL-6 secretion from iMos and pMos after 24h culture; *p<0.05, **p<0.01, ***p<0.001, ****p<0.0001; Scale bar=200 μm. (Statistical analysis: Mann-whitney U test for B, n=60, repeated thrice; ANOVA for C and D, n=3, repeated thrice). [file 12951_2022_1751_MOESM2_ESM.jpg]

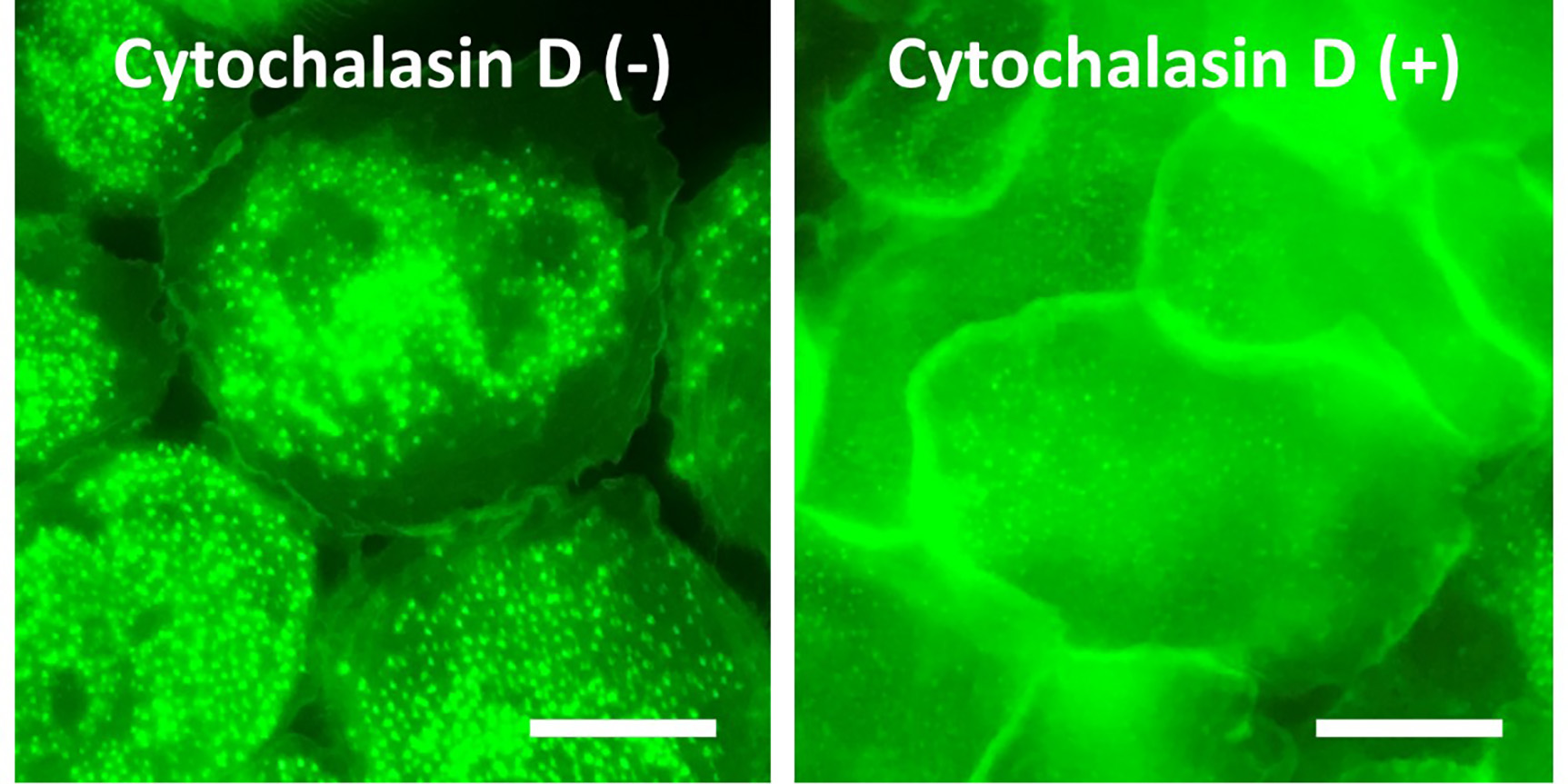

Supplement: Supplementary file 3 — Additional file 3: Fig. S3. The F-actin staining (FITC-Phalloidin) of iMos. Left panel: iMos in absence of Cytochalasin D; Right panel: iMos in presence of Cytochalasin D (200 ng/ml) after 12 h. Scale bar=25 μm. [file 12951_2022_1751_MOESM3_ESM.jpg]
